# Optical Screening Methods for Pesticide Residue Detection in Food Matrices: Advances and Emerging Analytical Trends

**DOI:** 10.3390/foods10010088

**Published:** 2021-01-05

**Authors:** Aristeidis S. Tsagkaris, Jana Pulkrabova, Jana Hajslova

**Affiliations:** Department of Food Analysis and Nutrition, Faculty of Food and Biochemical Technology, University of Chemistry and Technology Prague, Technická 5, Prague 6—Dejvice, 166 28 Prague, Czech Republic; pulkrabj@vscht.cz (J.P.); hajslovj@vscht.cz (J.H.)

**Keywords:** pesticide residues, optical detection, screening methods, point-of-care diagnostics, smartphones, biosensors, bioassays, food

## Abstract

Pesticides have been extensively used in agriculture to protect crops and enhance their yields, indicating the need to monitor for their toxic residues in foodstuff. To achieve that, chromatographic methods coupled to mass spectrometry is the common analytical approach, combining low limits of detection, wide linear ranges, and high accuracy. However, these methods are also quite expensive, time-consuming, and require highly skilled personnel, indicating the need to seek for alternatives providing simple, low-cost, rapid, and on-site results. In this study, we critically review the available screening methods for pesticide residues on the basis of optical detection during the period 2016–2020. Optical biosensors are commonly miniaturized analytical platforms introducing the point-of-care (POC) era in the field. Various optical detection principles have been utilized, namely, colorimetry, fluorescence (FL), surface plasmon resonance (SPR), and surface enhanced Raman spectroscopy (SERS). Nanomaterials can significantly enhance optical detection performance and handheld platforms, for example, handheld SERS devices can revolutionize testing. The hyphenation of optical assays to smartphones is also underlined as it enables unprecedented features such as one-click results using smartphone apps or online result communication. All in all, despite being in an early stage facing several challenges, i.e., long sample preparation protocols or interphone variation results, such POC diagnostics pave a new road into the food safety field in which analysis cost will be reduced and a more intensive testing will be achieved.

## 1. Introduction

The ever-increasing demand for food production unfortunately still requires a widespread use of pesticides. According to the European Commission (EC), pesticides “prevent, destroy, or control a harmful organism (“pest”) or disease, or protect plants or plant products during production, storage, and transport”. Pesticides can be clustered on the basis of the target pest (Table 1), for example, compounds combating insects are called insecticides [1]. Another useful classification was proposed by the World Health Organization (WHO) and is based on hazard expressed as lethal dose (LD) in rat specimen (Table 1) [2]. Alternatively, pesticides can be classified focusing on how they enter into the target pest, for instance, systemic pesticides are absorbed by tissues (leaves, roots, etc.) (Table 1) [3].

Regardless their classification, pesticide residues are related to toxicity issues, which can be either acute or chronic. The various pesticide classes can potentially affect their targets in different ways, including humans. In the case of organochlorine (OC) pesticides, which were extensively used during the 20th century, nervous system stimulation has been noticed. For example, lindane inhibits the calcium ion influx and Ca- and Mg-ATPase, causing release of neurotransmitters [4] and acting as a hormone disruptor causing both acute and chronic adverse effects ranging from dermal irritation or headache to cancer, Parkinson’s disease, or deficit immune system [5]. In the case of carbamate (CM) and organophosphate (OP) insecticides, their toxicity is related to the inhibition of acetylcholinesterase (AChE), a vital enzyme in the neural system of insects or mammals, including humans. Normally, AChE hydrolyzes the neurotransmitter acetylcholine into choline and acetic acid, an essential reaction that enables the cholinergic neuron to return to its resting state after activation. However, AChE activity is reduced in the presence of CMs and OPs due to carbamylation or phosphorylation of the serine hydroxyl group in the enzyme active cite [6], respectively. This results in acetylcholine accumulation, which can lead to serious health problems, including respiratory and myocardial malfunctions [7]. Another example of pesticide toxicity it is the class of pyrethroid pesticides. Pyrethroids cause neuronal hyperexcitation, resulting in repetitive synaptic firing and persistent depolarization. Their molecular targets are similar in mammals and insects, and include voltage-gated sodium, chloride, and calcium channels; nicotinic acetylcholine receptors; and intercellular gap junctions [8]. Therefore, it is obvious that the presence of pesticide residues in food has to be strictly regulated and monitored to protect consumer health.

To achieve that, accurate, sensitive, and robust analytical methods are of indispensable importance to assure that pesticide residues in food matrices are efficiently controlled. Liquid chromatography–tandem mass spectrometry (LC–MS/MS) and gas chromatography–tandem mass spectrometry (GC–MS/MS) are commonly applied [9,10] in various matrices, e.g., fruits and vegetables [11], honey [12], rice [13], and food of animal origin [14], enabling wide linear ranges and limits of detection (LODs) down to the µg kg^−1^ level. The use of triple quadrupole (QqQ) as the mass analyzer operating in the selected reaction monitoring (SRM) mode is the common way to detect for pesticide residues. However, at least two product ions are necessary for a compound identification while the ion ratio from sample extracts should be within ±30% of calibration standards from the same sequence (SANTE/12682/2019 guideline). Therefore, this requirement highlights a major drawback of SRM mode as the more pesticides included in the method, the more the necessary ion transitions that have to be measured. Thus, there is an increased chance of common or overlapped transitions affecting the method detectability [15]. To counter this problem, high-resolution mass spectrometry (HRMS) targeted methods have been proposed as an alternative [16,17,18]. Orbitrap, time-of-flight (TOF), and hybrid analyzers such as quadrupole-Orbirtap (q-Orbitrap) and quadrupole-TOF (qTOF) are used as the mass detectors, providing accurate mass measurement (<5 ppm), high resolution (more than 20,000 full width at half maximum (FWHM)), structural elucidation, and full MS scan capabilities (usually for the range 100–1000 Da). HRMS detectors resolve SRM-related problems, but there is still controversy on their quantification capabilities in comparison to QqQ methods. In any case, although chromatographic methods coupled to MS detectors provide the aforementioned merits, they are also time-consuming, laborious, and expensive methods that cannot be applicable by any laboratory around the world. Consequently, it is necessary to seek for alternatives able to combine sufficient detectability with cost-efficiency, simplicity, and applicability at the point of need.

In this way, screening methods have been introduced in food contaminant analysis featuring a great potential [9]. According to the Decision 2002/657/EC, “screening methods are used to detect the presence of a substance or class of substances at the level of interest”. There are several methods fitting within this concept aiming to achieve rapid, selective, cost-efficient, and sensitive screening in the food safety field [19]. Such methods are usually based on bio-affinity interactions between selective biomolecules, e.g., antibodies [20] or enzymes [21], and pesticide residues, while biorecognition events are typically monitored by either optical or electrochemical transducers [22]. In fact, optical transduction systems correlate biorecognition events to a color development/change, indicating their user-friendliness. The potential of such optical screening methods can be enhanced by coupling them with smartphones to achieve ubiquitous biosensing [23]. As we comprehensively discussed in our recent study [24], unprecedented characteristics have been introduced into chemical analysis due to smartphones, such as online results or end-user implementation, and this can obviously impact pesticide residue analysis as well.

In this study, a comprehensive overview on optical screening methods used in pesticide residue analysis is presented, focusing on the period 2016–2020. To identify the analytical performance that screening methods need to attain, we provide a critical discussion on EU regulatory framework. In fact, pesticide residues set two great challenges that need to be urgently faced. Firstly, pesticide regulatory limits are quite low (see Section 3), meaning that the developed screening methods need to demonstrate sufficient detectability into food extracts. Secondly, multi-step sample preparation protocols are commonly utilized (see Section 4.1), increasing the total analysis time and eliminating the advantage of rapid analysis provided by screening methods. Last but not least, the emergence of smartphones as analytical detectors is discussed, highlighting the novel capabilities brought by this technology in the field.

## 2. Pesticide Residue Occurrence in Food Distributed in the EU

**Figure 1:**
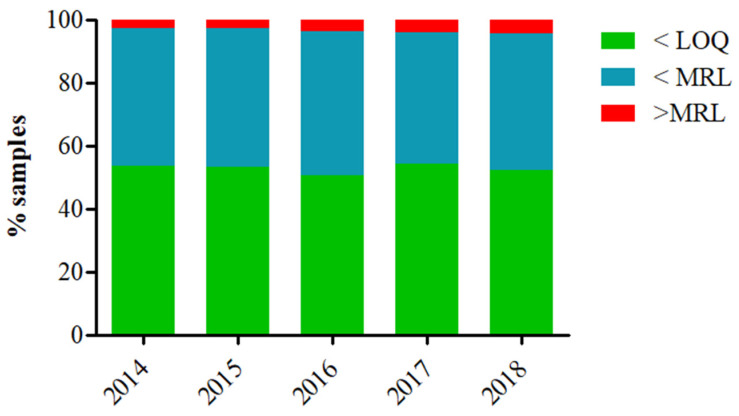
Temporal evaluation of the percentage samples that contained (i) no quantifiable residues (<limit of quantification, LOQ), (ii) residues at or below maximum residue levels (MRLs), and (iii) residues at a higher concentration than MRLs. The depicted data are extracted from the official EU reports on pesticide residues in food [25,26,27,28,29].

The European Food Safety Authority (EFSA) compiles yearly the EU report on pesticide residues in food, which contains data from the EU countries as well as Iceland and Norway. Therefore, pesticide residue monitoring is systematically performed, and a clear view of the applied testing is available. On the basis of the latest available data from the official EU reports [25,26,27,28,29], the vast majority of tested samples (always more than 95% of the samples, Figure 1) fell below the maximum residue levels (MRLs). However, although the tested samples were complied with regulatory requirements, there was a minor tendency of more samples be non-compliant during the last five reported years. In fact, the number of samples with non-quantifiable residues or contained residues within the legally permitted levels dropped from 97.1% in 2014 to 95.5% in 2018. This is likely related to (i) the slightly increased tested samples (about 83,000 samples were tested in 2014 while 91,000 samples were tested in 2018) and (ii) the globalization of food market, resulting in increased food imports from countries with different regulatory requirements. Worth noticing is that samples containing non-quantifiable amounts of pesticide residues are transferred to the labs and analyzed by expensive and time-consuming chromatographic methods underpinning the importance to implement screening methods into residue controlling. Obviously, the use of screening methods aims to assist instrumental analysis, resulting in rapid results and a better utilization of available recourses. Significantly, CM and OP residues have been commonly detected or even exceeded the MRLs. In fact, chlorpyrifos, carbofuran, dimethoate, acephate, profenofos, methomyl, methamidophos, and ethephon (all CM and OP insecticides) residues were among the compounds with the most frequent MRL exceedances [25,26,27,28,29]. Chlorpyrifos, an OP compound, was steadily within the top five pesticide residues with the most exceedances (except in 2017, when it was reported in ninth place), whilst in the latest report, chlorpyrifos was the compound with the most exceedances of its acute reference dose (ARfD). In this way, an official ban has been recently applied in the EU due to concerns predominantly related to neurotoxicity issues [30]. This fact can also explain why there is a variety of screening methods measuring CM and OP residues (see Section 4.2).

## 3. EU regulatory Requirements on Pesticide Residues

The EU regulatory framework related to pesticide residues is comprehensively set. In detail, MRLs for about 1100 pesticides in 300 different matrices has been established according to the EC Regulation 396/2005. To navigate and find the regulatory limits for a selected analyte, an online database has been developed permitting regulatory levels export in an excel file format (https://ec.europa.eu/food/plant/pesticides/eu-pesticides-database/mrls/?event=search.pr, last accessed 23 December 2020). However, although EU MRLs are established for unprocessed food, there are no EU MRLs for processed or composite foodstuffs. The Food and Agriculture Organization of the United Nations and the World Health Organization (FAO/WHO) have included MRLs for selected processed food in the Codex Alimentarius (http://www.fao.org/fao-who-codexalimentarius/codex-texts/dbs/pestres/commodities/en/, last accessed 18 August 2020). A similar approach has also been followed by the German Federal Institute for Risk Assessment (BfR), which provides an online tool for MRL calculation in processed food (https://www.bfr.bund.de/cm/349/bfr-compilation-of-processing-factors.xlsx, last accessed 18 May 2020). In case that there is no MRL for a pesticide, then a default 0.010 mg kg^−1^ limit is set; moreover, the default MRL is also used for infant food according to the Directive 2006/141. Infants (up to 12 months old) and young children (1 to 3 years old) are quite sensitive towards residues since their body weight is low and they face a greater risk when consuming a contaminant compared to an adult individual. Regarding the cumulative risk assessment, this is a major issue since the MRLs are prescribed for single residues, but food may be contaminated with multiple pesticide residues. In this context, a large amount of effort has been devoted to establish guidelines and a step towards this direction was an online tool called “Acropolis” developed by the National Institute for Public Health and the Environment for the Netherlands (RIVM) [31]. It is noteworthy that although the EFSA cannot set any regulatory requirements, its opinion is highly anticipated by the European Commission to prescribe any regulations. Undoubtedly, the legislation application is directly linked to the analytical capabilities and the quality assurance of the provided results.

## 4. Pesticide Residue Optical Screening in Food Matrices

The detection of pesticide residues is a great analytical challenge considering their diverse physicochemical characteristics and the numerous combinations of analyte-matrix. In addition, using optical screening methods pose further challenges, as in contrast to instrumental analysis, such methods sometimes face specificity, sensitivity, or robustness problems. In the following paragraphs, a critical discussion on sample preparation, optical screening methods, and their coupling to smartphones is provided to monitor the readiness of this upcoming technology in the pesticide residue analysis.

### 4.1. Sample Preparation

**Figure 2:**
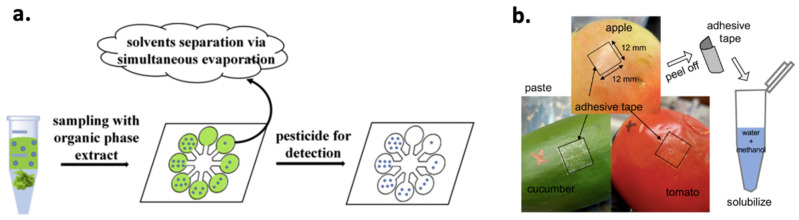
(**a**) Paper-based organic solvent evaporation for pesticide residue screening using enzymatic recognition. Reproduced with permission from [32]. (**b**) A simple and cost-efficient sample preparation protocol using an adhesive tape and a water–methanol solution to extract pesticides from fruit and vegetable peels. Reprinted with permission from [33]. Copyright 2020 American Chemical Society.

Sample preparation is a key step towards specific, sensitive, and accurate detection of pesticide residues. In the case of screening methods, high-throughput (in terms of tested samples) and short analysis duration need to be achieved while detectability should also be satisfactory (attained LODs lower than MRLs). Nevertheless, pesticide residues are commonly extracted using organic solvents and long sample preparation protocols. This is a major challenge for screening methods as they usually exploit selective biomolecules that have certain tolerance towards organic solvents (typically used as pesticide residue extractants). In fact, after a certain organic solvent content (commonly 20–30%) biomolecules are denaturized and lose their functionality, for example catalytic activity in the case of enzymes. Therefore, there have been efforts to extract pesticide residues using aqueous buffers, e.g., phosphate-buffered saline (PBS), since such solutions can adjust the pH value, which is vital for the proper biomolecule function. Sample incubation or mixing with a buffer, followed by a filtration to reduce matrix interferent compounds is a simple procedure that can be applied when using screening methods. Obviously, accuracy and/or detectability can be affected by such simplified sample preparation (due to co-isolated matrix compounds), underlying the need for highly selective recognition elements. It is worth noting that the emergence of paper analytical devices can provide a solution in this problem. Paper matrix can be used as an evaporation platform due to its large specific surface enabling air–liquid contact, which speeds up organic solvent evaporation easily [32] (Figure 2a). Therefore, extraction using organic solvents followed by paper-based solvent evaporation and then addition of the recognition element can be applied to face this challenge. Another practical and cost-efficient solution was recently published [33], in which adhesive tape (Figure 2b) was stuck to a vegetable surface, peeled off, and dipped into a water–methanol solution achieving a LOD around 0.20 μM (0.066 mg kg^−^^1^) for malathion depending the tested matrix. In any case, there are still screening methods that use sample preparation protocols commonly applied in instrumental analysis, for example, quick easy cheap effective rugged and safe (QuEChERS) extraction [34,35] to achieve a better analytical performance. Unfortunately, the use of multi-step sample preparation protocols in pesticide residue screening methods remains a bottleneck.

### 4.2. Optical Screening Methods

#### 4.2.1. Biochemical Assays

**Figure 3:**
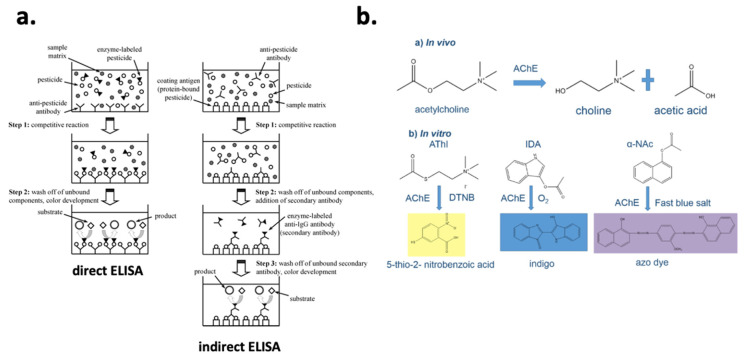
(**a**) Multistep direct and indirect ELISA protocols for pesticide residues screening. Reprinted with permission from [47]. Copyright 2013 American Chemical Society. (**b**) In vivo and in vitro acetylcholinesterase hydrolytic activity producing, in vitro, various colored products depending the catalyzed substrate. Reprinted from [42] under CC BY 4.0.

Biochemical assays using antibodies or enzymes as recognition elements have been traditionally used in a microplate format, which provides high-throughput, simplicity, good sensitivity, and ease of operation. The enzyme-linked immunosorbent assay (ELISA) is a striking example of such bioassays. ELISA is based on the specific interaction between an enzyme-labelled analyte-specific antibody and its antigen. Owing to the labelling of the antibody with an enzyme, upon the addition of a substrate, a measurable color change is initiated. A recent review by Wu et al. [36] is recommended for a deeper understanding of the ELISA mechanism, various types (Figure 3a), as well as recent advances. ELISAs have been developed for the screening of various pesticide residues in food matrices, for example, OPs [37,38], CMs [39], neonicotinoids [40], or fungicides [41]. In terms of cholinesterase microplate assays, cholinesterases have been employed as recognition elements (both AChE [42] and butyrylcholinesterase, BChE [43]) to screen for CM and OP. Considering that, in vitro, cholinesterases hydrolase colorless substrates to colored products, the presence of CMs and OPs can be correlated to a color decrease similarly to competitive ELISAs. A great variety of substrates, resulting in different colored products (Figure 3b), have been used including acetylthiocholine and butyrylthiocholine halides for AChE and BChE, respectively; indoxyl acetate; α-naphthyl acetate; 2,6-dichloroindophenol acetate; and others [44]. Importantly, reduced sample and reagent consumption (typically less than 100 μL) as well as low LODs at the μg kg^−1^ level [42,45,46], depending on the matrix, were achieved by cholinesterase microplate assays. However, biochemical assays are still applicable in laboratories as they require certain apparatus and well-trained operators (commonly such assays contain multiple steps).

#### 4.2.2. Biosensors

Biosensors are analytical platforms that convert a biological response into a quantifiable and processable signal. Besides the described attractive characteristics of biochemical assays, biosensors can be miniaturized and automated, indicating their potential for on-site testing. On the basis of the biorecognition element, we can distinguish three main groups of biosensors, i.e., immunosensors [20], cholinesterase [21] and lipase sensors [48] (enzymatic recognition), and aptasensors [49,50]. It is of note that aptamers emerge as an alternative to counter problems related to antibodies, such as the challenge to trigger an immune response for small molecules or their higher temperature stability, a problem related to biomolecules [51]. Biomolecules can be negatively affected by organic solvents (e.g., denaturation problems resulting in decreased activity), certain pH values (commonly neutral pH values are the optimum for antibodies and enzymes), or hydrostatic and osmotic pressure. Nevertheless, increased stability can be accomplished by immobilizing biomolecules on surfaces as in the case of biosensors [52]. For instance, the immobilization of AChE on cellulose strips resulted in retained enzyme activity over a two-month period [34]. Other less used recognition elements include, but are not limited to, molecularly imprinted polymers (MIPs, synthetic molecules), cells, and DNA probes. In the following paragraphs, further discussion on various biosensors is provided on the basis of the detection principle used, and tables summarizing interesting publications in the field during the period 2016–2020 are presented.

##### Colorimetric Biosensors

Colorimetry is probably the simplest approach as a biorecognition event is related to a color development. This fact significantly increases colorimetric platforms potential for on-site analysis as colorimetric signals can be monitored even by the naked eye or they can be easily coupled to a smartphone readout (see Section 4.3). On the downside, colorimetric signals are vulnerable to minor lighting variations while most of the food extracts are colored, which negatively effects method detectability. Of importance is the ever-increased use of analytical platforms commonly based on colorimetric responses such as membrane-based assays (lateral flow (LF) or paper-based assays), microfluidic chips, or lab-on-a-chip (LOC) devices (Table 2). LF assays are membrane tests consisting of various polymeric zones on which various substances can be accommodated and react with an analyte [53]. Liquid samples or extracts containing an analyte move through this lateral device due to capillary forces. Two different formats of LF assays can be distinguished, namely, competitive and sandwich formats. Competitive assays are used for low molecular weight analytes, i.e., pesticide residues, and a positive result is related to the absence of a test line due to the blocking of antibody binding sites to protein conjugates by the analyte. In terms of big molecules, for example, allergens, the sandwich format is used, and the analyte is immobilized between two complementary antibodies. Besides research studies using LF assays for pesticide residue screening [54,55], LF assays are one of the few cases that have reached the commercialization stage [19]. Regarding microfluidics, this is a relatively new field that was established in 2006 following the publication of G.M Whitesides in the prestigious *Nature* journal [56]. In this way, microfluidics are related to the manipulation of fluids in channels with dimensions of tens of micrometers. Fluidic behavior under these micro-level confined regions significantly differs from fluidic behavior in the macroscale. In this context, essential parameters such as viscosity, density, and pressure need to be strictly controlled to reach optimum microfluidic performances [57]. Although no strict criteria have been proposed to define microfluidic systems, the length and internal size of the channels is considered of critical importance. Microfluidic channels are combined to LOC devices to develop fully portable and autonomous analytical platforms. In fact, LOC systems are able to mimic different apparatus such as reactors and pumps to carry out injection, filtration, dilution, and detection in a reduced portion, eliminating handling errors and enhancing robustness while retaining the analysis cost low [58]. Regarding the application of colorimetric microfluidic and LOC platforms, paper-based microfluidics can combat problems related to intolerance towards organic solvents that are used to extract pesticide residues by spontaneous evaporation on the paper-platform before loading an enzyme solution for pesticide recognition [32]. However, overall, such platforms are still in an early stage, with the majority of the studies focusing on proof-of-concept applications [59]. Unfortunately, the majority of colorimetric analytical platforms utilize traditional sample preparation protocols, highlighting the need to automate and simplify sample pretreatment to increase the applicability of such methods in the field.

##### Fluorescent Biosensors

Biosensors with fluorescent detection combine the selectivity provided by the recognition part to the sensitivity of fluorescence (FL), as it is a zero-background method and only specific compounds (based on their structure) are able to fluoresce. Fluorescent biosensors (Table 3) are based on the principle that the interaction of a fluorescent probe (chemical or physical) with an analyte leads to either fluorescence enhancement or quenching [66], which is also known as analyte-induced “on–off” fluorescent behavior [67]. A great variety of fluorescent probes have been used, namely, fluorescent dyes, nanocomposite materials, rare earth elements, or semiconductors [68]. The great advancements in nanomaterial field have further improved fluorescent detection, as they have countered, at a certain extent, bottlenecks related to dyes, e.g., high photobleaching. Quantum dots, which are semiconductor crystalline nanomaterials with unique optical properties due to quantum confinement effects, are an example of nanocomposite probes that have enhanced fluorescent detection for pesticide residue screening [66]. This was recently demonstrated for the detection of four OP pesticides, namely, paraoxon, dichlorvos, malathion, and triazophos, using CdTe quantum dots as the fluorescent probe coupled to an AChE-choline oxidase enzyme system [69]. In this case, when AChE was active (resulting in choline production), H_2_O_2_ was produced by choline oxidase, which in turn “turned off” the FL of the CdTe quantum dots. However, in the presence of an OP, the FL induced by CdTe quantum dots was retained and a correlation between OP concentration and FL signal was feasible. Impressively, a LOD of 0.5 ng mL^−1^ was achieved in water, tomato juice, and apple juice, while the fluorescent biosensor could be regenerated using pyridine oximate. In another study, an “off−on−off” strategy was applied by using AChE as the recognition element and lanthanide-doped upconversion nanoparticles (UCNPs) with Cu^+2^ as the fluorescent probe [70]. This analytical platform achieved an LOD of 0.005 mg kg^−1^ for diazinon detection in apple and tea powder and, importantly, the results were cross-confirmed to GC–MS. It should be kept in mind that although it is necessary to benchmark the results attained using screening methods, this practice is commonly omitted in the published literature as it is comprehensively discussed in our previous study [9]. In conclusion, FL biosensors can attain sensitive results, which is extremely important in the food safety field. However, their principles and analytical configuration are commonly more complicated than colorimetric platforms that may influence their applicability within the point-of-care (POC) testing concept.

##### Surface Plasmon Resonance Biosensors

Surface plasmon resonance (SPR) biosensors are based on an optical phenomenon that happens on a thin conducting film at the interface between media of different refractive index [78]. SPR provides label-free sensing, which is a great advantage as labeling procedures are omitted, resulting in reduced cost and prevention against false positive signals related to labeling. Moreover, SPR is especially useful to calculate association (or dissociation) kinetics and affinity constants or bounded analyte content in the case of immunorecognition [79]. Interestingly, only a few enzyme-based biosensors have employed SPR detection [80]. Detecting pesticide residues in trace amounts is a challenging task as it is difficult to attain a measurable change in the refractive index due to their low molecular mass. To face this problem, sensor surface modification using nanoparticles is commonly applied since nanomaterials can enhance SPR signals due to their high refractive index. Furthermore, nanomaterials are also preferred because of their facile synthesis, high surface to volume ratio, and high biocompatibility and photostability [81]. The nanomaterials commonly utilized in such analytical platforms include, but are not limited to, metal nanoparticles, i.e., Au or Ag; carbon nanoparticles; and quantum dots. Besides signal enhancement using nanomaterials, SPR phase-measurement instead of amplitude (which is the case in conventional SPR systems) is an alternative approach that is based on the topological nature of the phase of a system. Considering that our study focuses on the analytical developments and applications in pesticide residue analysis, no further discussion on the physics behind phase sensitive SPR measurement is provided, and two studies [82,83] are recommended for a deeper understanding of the phenomenon. In any case, SPR biosensors have found several applications in pesticide residue analysis based mainly on immunorecognition (Table 4). It can be noticed that the problem of laborious sample preparation when analyzing solid food matrices was also the case for SPR-based biosensors. In addition, the low molecular weight of pesticides set a great challenge in terms of detectability and compliance to regulatory limits for SPR-based analytical platforms. More effort is definitely needed to further improve such platforms, considering the miniaturization potential (handheld SPR systems or coupling to smartphones) [84] that can be highly beneficial for the field.

##### Surface-Enhanced Raman Spectroscopy

Although some consider surface-enhanced Raman spectroscopy (SERS) as an optical biosensor due to its coupling to biorecognition events [20], SERS is in principle a spectroscopic method based on light scattering, specifically to inelastic collisions occurring between a sample and incident photons emitted by a monochromatic light source, such as a laser beam [91]. Combining biorecognition events to SERS can significantly enhance the analytical performance of such methods, but also it increases method complexity and cost. For example, a multiplexed immunochromatographic assay for the simultaneous detection of cypermethrin and esfenvalerate (pyrethroid pesticides) achieved impressive results in milk matrix [92]. Specifically, the acquired LOD was at the parts per trillion level (LOD = 0.005 ng mL^−1^), a performance that would not be possible without using SERS-based detection considering that immunochromatographic assays mostly provide qualitative results. Regarding direct SERS screening, this is feasible as molecules provide specific Raman spectra due to their unique structure, which is also called “Raman fingerprint”. However, Raman signals are not strong enough, with only 1 out of 10 million of the scattered photons experiencing Raman scattering when incident light interacts with an analyte [93]. Therefore, it is necessary to enhance such signals by employing nanocomposite substrates resulting in electromagnetic and chemical enhancement [94]. Two different types of substrates can be distinguished, namely, colloidal and solid substrates. Although the synthesis of colloidal substrates such as Ag or Au nanoparticles is quite facile and cost-effective, poor reproducibility of signals remains a problem [95]. In terms of solid substrates, these provide more robust signals and counter the risk of nanoparticle aggregation, which is a problem for colloidal substrates. Solid substrates can be immobilized on various surfaces for example paper [96] or hydrogels [97]. In fact, paper-based SERS substrates can further increase the method potential to be applied on-site as such substrates can be used to swab the surface of a sample and then screen using a portable Raman spectrometer. In this way, paper SERS substrate coated with a monolayer of core-shell nanospheres was recently developed and was successfully used for the detection of thiram in orange juice [98]. This simple and non-destructive method achieved a LOD of 0.25 μM or 0.060 mg L^−1^ by using 4-methylthiobenzoic acid (4-MBA) as the internal standard (IS) to attain quantitative results. Similarly, in another study, 4-MBA was accommodated in Au@Ag nanocubes and exploited as the IS [99]. Moreover, it was noticed that water molecules can be used as a IS since their Raman scattering signal is quite stable [100]. Alternatively, the use of anisotropic nanoparticles, e.g., nanocubes, nanorods, and nanostars, positively affected SERS quantification capabilities by achieving more stable signals [101]. Nevertheless, SERS can mostly detect analytes on the surface of food, which does not correspond to the whole amount of a pesticide in a food matrix. Pesticide residues depending their polarity can be found in the non-polar peel or the polar-aquatic inner part of a fruit. Moreover, LODs have been mostly expressed using the “ng cm^−2^” unit [102] because pesticide residues were measured on a surface. Nevertheless, such a concentration expression is not in line to the regulated MRL units (mg kg^−1^). There were also cases in which QuEChERS extraction [103] or other long sample preparation protocols (Table 5) were used prior to SERS screening, an approach that comes in contrast to the non-destructive and direct measurements than can be acquired using SERS. In conclusion, SERS can highly improve the current status of pesticide residue screening at the point of need due to the discussed merits and the ever-decreased price of such portable platforms (approximately EUR 35,000 to 50,000 at the moment).

### 4.3. Coupling Optical Screening Methods to Smartphones

As already discussed in the previous paragraphs, the analytical signal of optical screening methods, especially in the case of colorimetry, is a simple and user-friendly indication of pesticide residue presence in food matrices. In terms of biochemical assays, such signals are commonly monitored using benchtop instruments, for example, absorbance readers, to acquire semi-quantitative or quantitative data. Regarding biosensors, these analytical platforms can also be handheld, providing on-site results, which can be extremely useful for detecting pesticide residues in imported foodstuff at the control point, i.e., border controls or at the field testing. Nevertheless, optical biosensors usually attain either qualitative results on the basis of visual inspection of the tested assay or semiquantitative results using readers, e.g., readers for LF assays, which significantly decrease the portability potential of such analytical platforms.

To face this challenge and introduce further unprecedented characteristics, smartphones have emerged as an alternative analytical detector combined to bioassays [23,110]. In principle, smartphone camera can be used as an optical biosensor to record images or videos containing the analytical useful information, enabling result semi-quantitation. Moreover, on-site one-click results exploiting smartphone computing power are feasible using smartphone apps. Interestingly, these results can be instantly communicated due to the online connectivity provided by smartphones as well as geo-located, potentially creating heatmaps during an outbreak situation. Such an option could be extremely useful during the fipronil insecticide scandal in 2017 (https://edition.cnn.com/2017/08/10/health/europe-egg-scandal-contamination-arrests/index.html, last accessed 8 November 2020), when egg farms in the Netherlands violated the regulatory limits and supplied contaminated eggs in the EU market. Actually, the available analytical scheme posed itself a key challenge during the fipronil scandal. In detail, samples needed to be collected; transported; marked with a unique laboratory code to assure traceability; and finally analyzed using instrumental analysis, in this case chromatographic methods [111]. The response in this health threat for the EU consumers would be totally different if smartphone assays were available at that moment. Smartphone assays could be used for an initial on-site screening, omitting the collection and transportation steps, generating instantly a sample ID, and providing a screening result with a certain false positive/false negative rate. In other words, smartphone-based analysis can assist the current analytical scheme by accelerating processes and sending only suspected samples to the lab.

Unfortunately, smartphone-based analysis has not yet reached such a technology readiness level (TRL) to be actively implemented into the analytical scheme. The majority of studies focus on proof-of-concept results (Table 6), with insufficient application on food matrices, especially in the case of solid food [24]. This is mostly related to the laborious sample preparation protocols that are necessary to extract pesticides from food matrices, mostly fruits and vegetables. Obviously, combining pocket-sized analytical platforms to laboratory protocols minimizes their actual portability potential and drives the field to the so called “chip-in-a-lab” era [112]. Chip-in-a-lab is a term used to describe the development of POC platforms that are unable to operate without the complementary use of certain laboratory equipment. In our view, the development of micro total analysis systems (μTAS) enabling integrated sample preparation is a necessity for field-ready and consumer-focused diagnostics [113]. To date, there is a lack of such systems, especially in the case of solid food matrices for the vast majority of analytes. Recently, a smartphone-based platform providing a sampling-to-result solution was developed for multiplex allergen detection in cookies [114]. This platform integrates a completed analytical protocol on the device, which can be even applied by non-experts following simple instructions. Undoubtedly, such an approach paves the road for smartphone diagnostics in food analysis. Additionally, the use of prototype 3D-printed apparatus pinpoints the significance of implementing 3D printing into chemical analysis. Another significant bottleneck is result ruggedness when using different smartphone models. Indeed, smartphone-based analytical platforms are mostly coupled to a specific device questioning whether comparable results can be obtained with a different smartphone model [115]. In terms of the analytical signal used in smartphone-based optical assays, various approaches have been utilized, specifically the RGB color space [43], other color spaces (i.e., HSV or CIE-Lab) [116], and random combination of color spaces based on algorithms [117] or barcodes [118]. In general, there has not been a clear conclusion on which is the most useful approach, but RGB is the smartphone primary color space and thus can be directly used without the need of mathematical transformation as in the case of other color spaces. It is also unclear as to whether it is necessary to use auxiliary attachable parts such as 3D-printed elements [34] to standardize optical conditions or record under ambient light using correction algorithms [119]. Overall, smartphone-based pesticide residue analysis is at an early stage and further developments are definitely expected, indicating this technology potential to revolutionize the field.

## 5. Conclusions

A critical overview of the developed optical methods for pesticide residue screening is comprehensively presented. Importantly, yearly reports on the occurrence of pesticide residues in the food chain as well as well-established regulation are available in the EU. Pesticide residue monitoring and control are strictly related to the available analytical methods, which need to attain low LODs, high accuracy, and ruggedness. These performance characteristics are provided up to date by chromatographic methods coupled to MS detectors. However, there is an intensive research effort to establish more optical screening methods able to assist instrumental analysis and face challenges related to their high cost, laborious protocols, and necessity of highly trained users. Thus, various biochemical assays and biosensors based on optical detection have been developed during the last five years. Sample preparation using common laboratory protocols, for example, QuEChERS, remains a bottleneck that limits the current applicability of POC screening methods, indicating the need to develop fully integrated μTAS. Nevertheless, sometimes such protocols are the only way to satisfactory extract pesticide residues from complicated food matrices. Assay sensitivity and selectivity are critical performance characteristics that need to be always assessed. In this way, LODs must be attained in the tested food matrix and not in buffer solutions, which was the case in few cases. Acquiring LODs in buffer is useful during method optimization to monitor the optimum assay performance and test parameters, for example, enzyme substrate concentration. In terms of assay selectivity, this is also a crucial performance characteristic as biorecognition elements may be affected by other compounds with structure similar to analytes. A characteristic example of this is AChE, an enzyme widely utilized in bioanalytical methods for pesticide residue screening. Although both CM and OP pesticides inhibit AChE activity, their inhibitory potency highly varies depending on their structure. Therefore, cross-reactivity studies are of indispensable importance to monitor bio-affinity interactions and determine potential interfering compound effect on assay performance. Additionally, the absence of result confirmation using instrumental analysis is another challenge since screening results need to be verified. In terms of optical detection, colorimetry is the simplest and most user-friendly detection system, but FL, SPR, and SERS can usually provide more sensitive results due to their selectivity and combination to nanomaterials. In these cases, nanomaterials enhance the optical properties of detection systems proving their indispensable importance for POC diagnostics. Portable handheld SERS devices can further improve on-site pesticide residue detection at the point of need without the need of sample preparation. On-site screening can also be achieved by hyphenating optical screening assays to smartphones for ubiquitous sensing. Smartphone-based pesticide residue analysis can be extremely useful at border controls, considering the ever-increased globalization of the food market or at the field testing. To achieve that, however, sufficient detectability and a minimum false negative rate need to be achieved. Moreover, interphone result variation is a key parameter that has to be investigated more as most of the smartphone-based studies are applicable on a specific smartphone. In any case, the hyphenation of screening methods to smartphones is a step towards the “democratization” of chemical analysis and the introduction of new era, in which sensing is not strictly related to laboratories.

## Figures and Tables

**Table 1:** Summary of various classification systems for pesticides.

a. Based on Target Pest			
Pesticide Type		Pest	
Algicide		Algae	
Avicide		Birds	
Bactericide		Bacteria	
Fungicide		Fungi	
Herbicide		Weeds	
Insecticide		Insects	
Miticide		Mites	
Molluscicide		Snails	
Nematicide		Nematodes	
Piscicide		Fish	
Rodenticide		Rodents	
**b. Based on Toxicity**			
**Type**	**Toxicity Level**	**LD_50_ for Rats (mg kg^−1^ Body Weight)**	
		**Oral**	**Dermal**
Ia	extremely hazardous	<5	<50
Ib	highly hazardous	5 to 50	50–200
II	moderately hazardous	50–2000	200–2000
U	unlikely to present acute hazard	>5000	
**c. Based on the Way of Entry into a Pest**			
**Ways of Entry**		**Details**	
Systemic		Absorption by tissues such as leaves, stems, and roots	
Non-systemic		Physical contact between the pesticides and the target organism	
Stomach poisoning		Pesticide digestion	
Fumigants		Target organism killing through vapors	
Repellents		Inhibit the ability of pests to localize in crops	

**Table 2:** Selected studies on pesticide residue screening using colorimetric biosensors.

Analyte	Matrix	Analytical Platform	Sample Preparation	LOD	EU MRL	Reference
Methyl-paraoxon and chlorpyrifos-oxon	cabbage and dried mussel	paper-based device coated with nanoceria using an enzyme inhibition assay with AChE and ChOX	methanol vortex extraction, centrifugation, PSA clean-up, centrifugation, evaporation	0.040 mg kg^−1^	0.010 mg kg^−1^	[60]
Carbofuran and carbofuran-3-hydroxy	water	LF immunoassay	none	7 μg L^−1^ (carbofuran) and 10 μg L^−1^ (carbofuran-3-hydroxy)	0.1 μg L^−1^	[54]
Malathion	apple	aptasensor employing gold nanoparticles	methanol extraction, filtered and evaporation	5.2 pM (or 0.001 μg kg^−1^)	0.02 mg kg^−1^	[61]
Paraoxon	vegetable irrigation water	enzyme cascade and iodine starch color reaction	filtration	10 μg L^−1^	n.a.	[62]
Ethoprophos	tap water	gold nanoparticle aggregation combined to adenosine triphosphate	no	4 μM (or 0.96 mg L^−1^)	0.1 μg L^−1^	[63]
Paraoxon	rice and cabbage	AChE assay coupled to carbon dots	acetonitrile ultrasonic extraction, centrifugation, filtration through sodium sulfate and evaporation	0.005 mg kg^−1^	0.01 mg kg^−1^ (cabbage) and 0.02 kg^−1^ (rice)	[64]
Acetamiprid	spinach	aptamer with DNA probe	ethanol ultrasonic extraction, centrifugation, filtration, and 20-times dilution	0.1 nM (or 0.022 μg kg^−1^)	0.6 mg kg^−1^	[65]

**Table 3:** Selected studies on pesticide residue screening using fluorescent biosensors.

Analyte	Matrix	Analytical Platform	Sample Preparation	LOD	EU MRL	Reference
Acetamiprid	tea	aptasensor	methylene chloride extraction, filtration, and evaporation	0.002 mg kg^−1^	0.05 mg kg^−1^	[71]
Dichlorvos	cabbage and fruit juice	carbon dots–Cu(II) system	PBS extraction	0.84 ng mL^−1^	n.a.	[72]
Paraoxon	water	BChE assay	no	0.25 μg L^−1^	0.1 μg L^−1^	[73]
Imidacloprid	Chinese leek, sweet potato, and potato	LF immunoassay	PBS extraction and supernatant dilution with PBS	0.5 ng g^−1^	0.5 mg kg^−1^	[74]
Diazinon	cucumber and apple	aptasensor	Dilution with water, water-heated bath, centrifugation	0.13 nM (0.039 μg kg^−1^)	0.01 mg kg^−1^	[75]
Aldicarb	ginger	AChE-based assay	QuEChERS	100 μg kg^−1^	0.05 mg kg^−1^	[76]
Eight rodenticides	wheat	LF immunoassay combined with quantum dots	acetonitrile ultrasonic extraction, centrifugation, filtration, and filtrate 10-times dilution in PBS	1–100 μg kg^−1^ depending the analyte	0.01 mg kg^−1^	[77]

**Table 4:** Selected studies on pesticide residue screening using surface plasmon resonance (SPR) biosensors.

Analyte	Matrix	Analytical Platform	Sample Preparation	LOD	EU MRL	Reference
Parathion	cabbage washing solutions	AChE + SPR	The spiked cabbage sample was washed with 30 mL of distilled water twice	0.069 mg L^−1^	n.a.	[85]
Profenofos	water	fiber optic sensor based on MIP recognition	No sample preparation	0.02 μg L^−1^	0.1 μg L^−1^	[86]
Triazophos	cabbage, cucumber, apple	immunosensor	QuEChERS, 10-times dilution for cabbage and cucumber 20-times dilution for apple	0.1 μg kg^−1^ (cabbage and cucumber) and 0.4 μg kg^−1^	0.01 mg kg^−1^	[87]
Carbendazim	medlar	immunosensor with Au/Fe_3_O_4_ nanocomposite probe for SPR signal enhancement	80% methanol extraction, centrifugation, dilution with PBS to 5% methanol	5 ng mL^−1^ in the extract (there is no information about sample weight)	0.01 mg kg^−1^	[88]
Chlorothalonil	lettuce, cabbage, onion	immunosensor	Methanol extraction, centrifugation, 8.5 times dilution to 10% methanol	1 mg kg^−1^	0.6 mg kg^−1^ (cabbage) and 0.01 mg kg^−1^ (lettuce, onion)	[89]
Chlorpyrifos	maize, apple, cabbage, medlar	immunosensor	80% methanol extraction, supernatant diluted 10-times with PBS	0.0025 mg kg^−1^	0.01 mg kg ^−1^ (apple, cabbage, medlar) and 0.05 mg kg^−1^ (maize)	[90]

**Table 5:** Selected studies on pesticide residue screening using SERS methods.

Analyte	Matrix	Analytical Platform	Sample Preparation	LOD	EU MRL	Reference
Methyl parathion	apple	portable SERS	none	0.011 μg cm^−2^	0.010 mg kg^−1^	[102]
Prometryn and simetryn	wheat and rice	MIP-SERS	QuEChERS	20 μg·kg^−1^	0.010 mg kg^−1^	[103]
Thiram	lemon	SERS with nanowire Si paper as a substrate	none	72 ng cm^−2^	0.100 mg kg^−1^	[104]
Difenoconazole	pak choi	portable SERS	acetonitrile extraction, centrifugation, dSPE clean-up, evaporation, and reconstitution to ethyl acetate	0.41 mg kg^−1^	2.0 mg kg^−1^	[105]
Paraquat	apple and grape juice	portable SERS	none	100 nM (0.025 mg L^−1^)	n.a.	[106]
Dimethoate	olive leaves	portable SERS	none	5 × 10^−7^ M	n.a.	[107]
Edifenphos	rice	SERS	two times acetone extraction, centrifugation; six times pre-concentration	0.1 mg kg ^−1^	0.01 mg kg^−1^	[108]
Thiram	apple, pear, and grape	“drop-wipe-test” using portable SERS	none	5 ng cm^−2^	5 mg kg^−1^ (apple and pear) and 0.1 mg kg^−1^ (grape)	[109]

**Table 6:** Selected studies on pesticide residue screening using smartphone-based methods.

Analyte	Matrix	Analytical Platform	Sample Preparation	LOD	EU MRL	Reference
Chlorpyrifos, diazinon, and malathion	spinach, lettuce, and cabbage	LF multiplex aptasensor	homogenization and homogenate filtration	0.010 mg kg^−1^	0.01 to 0.5 mg kg^−1^, depending the analyte matrix	[120]
Carbofuran	apple	hybrid paper-LOC prototype	QuEChERS and evaporation	0.050 mg kg^−1^	0.001 mg kg^−1^	[34]
Chlorpyrifos methyl	cabbage	chemiluminescent enzyme origami paper-based biosensor	mixing with water and centrifugation	0.6 mM (193 mg kg^−1^)	0.01 mg kg^−1^	[121]
Acetochlor and fenpropathrin	corn, apple, and cabbage	multiplex LF immunoassay	PBS 0.05% Tween-20 and 10% methanol extraction, centrifugation, dilution	6.3 ng g^−1^ (acetochlor) and 2.4 ng g^−1^ (fenpropathin)	0.010 mg kg^−1^	[122]
Chlorpyrifos	fruit and vegetable wash water	lipase paper-based device		65 ng mL^−1^	n.a.	[55]
Methyl paraoxon	pear	nanoceria-based assay	ethyl acetate ultrasonic extraction, centrifugation, and evaporation	0.060 mg kg^−1^	0.010 mg kg^−1^	[123]
2,4-Dichlorophenoxyacetic acid	water	ELISA in 3D-printed device	no	1 μg L^−1^	0.1 μg L^−1^	[124]
